# Humanized mice as a useful model to study HIV-1 induced immune activation, its mechanisms and potential therapeutic approaches

**DOI:** 10.1186/1742-4690-8-S2-P62

**Published:** 2011-10-03

**Authors:** Maneesh Singh, Pratibha Singh, Dolores Vaira, Kjetil Tasken, Souad Rahmouni, Michel Moutschen

**Affiliations:** 1 Immunology and Infectious Diseases Unit, GIGA-R, University of Liege, Belgium; 2 CHU de Liege, Universite of Liege, AIDS Laboratory Reference, Liege, Belgium; 3 The Biotechnology Centre of Oslo, University of Oslo, Norway

## 

Recent understanding of HIV-1 pathogenesis mechanism has changed our views about possible mechanisms of CD4T-cell depletion during infection. Apart from HIV-1-mediated killing a more comprehensive explanation has appeared that includes T cell exhaustion and chronic immune activation as a central feature in HIV-1 pathogenesis. While highly active antiretroviral therapy (HAART) markedly reduces viral load, T cell activation levels and soluble markers of inflammation remain abnormally high. Markers of chronic activation, such as CD38, PD-1 or HLA-DR on T cells, appear to be better predictors for clinical progression during HIV infection than HIV RNA levels and CD4Tcell counts alone. Therefore, a better understanding of HIV-índuced immune activation and the design of new immunomodulatory approches in combination with HAART are needed.

We have generated an efficient model of human stem cells (HSCs) engraftment in NOD/LtsZ-scidlL-2R^null^ (NSG) mice that supports chronic HIV infection with high plasma viral loads. HIV-1 infection in these humanized mice is characterized by widespread immune activation with increased expression of PD-1, HLA-DR, CD38, CD69, CD25 and other immune activation markers. These humanized mice provide an effective *in vivo* system for the assessing novel approaches for their potential in suppressing chronic immune activation during HIV-1 infection, in absence of interference of antiretroviral therapy. In this study, we evaluated *in vivo* the benefits of two novel approaches aimed at reducing HIV-induced immune activation.

Minocycline is an antibiotic of the tetracycline family with anti-inflammatory and immunomodulatory properties affecting CD4 T cells activation by a mechanism involving the inhibition of the NF-AT1 transcription factor activity. We hypothesized that this antibiotic could suppress the HIV-1-induced chronic immune activation and thus, limit the HIV pathogenesis when combined to HAART. Therefore, we treated HIV-1 (JRCSF) infected-humanized NSG mice with minocycline (100mg/kg/day) for 60 days. We next evaluated the expression, by flow cytometry, of several T cells activation markers together with CD4^+^T cells counts. Our data suggest that minocycline is effective in suppressing HIV-1 induced immune activation in peripheral blood and lymphoid organs (spleen, lymph nodes and bone marrow). Levels of cellular immune activation markers such as PD-1, HLA-DR, CD38, CD69, CD25, CD28 and CTLA-4 were significantly lower in minocycline treated group. These immunological benefits of minocycline were correlated with higher CD4^+^T cell counts in the treated group.

The immune activation which is associated with retroviral infection is also associated with increased levels of intracytoplasmic cyclic AMP which could act as a positive feedback loop in the infection since several reports have suggested that cAMP and downstream signaling pathways play an important role in the permissivity of susceptible cells to HIV infection and replication. We have used a peptide which prevents the binding of the catalytic subunit of PKA type I to its anchoring protein and therefore blocks most effects of cyclic AMP within lymphocytes and monocytes (RIAD peptide). Mice were treated with 3.5 mg/kg of RIAD peptide weekly. Treatment of humanized mice with RIAD peptide limited viral replication after high dose of HIV intraperitoneal challenge and reduced the intracytoplasmic levels of cyclic AMP.

Further experiments are needed to better appreciate the therapeutic potential of these novel therapies in the suppression of HIV-induced chronic immune activation.

